# Androgen Receptor-Dependent Mechanisms Mediating Drug Resistance in Prostate Cancer

**DOI:** 10.3390/cancers13071534

**Published:** 2021-03-26

**Authors:** Marzieh Ehsani, Faith Oluwakemi David, Aria Baniahmad

**Affiliations:** Institute of Human Genetics, Jena University Hospital, Am Klinikum 1, 07740 Jena, Germany; m67.ehsani@gmail.com (M.E.); faith-oluwakemi.lena.david@uni-jena.de (F.O.D.)

**Keywords:** androgen receptor, prostate cancer, AR antagonists, castration resistant PCa, androgen deprivation therapy

## Abstract

**Simple Summary:**

Prostate cancer can develop under hormone treatment and chemotherapy from a castration-sensitive towards a castration-resistant into a drug resistant-tumor. The main hormonal drug target is the androgen receptor (AR). Androgen deprivation therapy reduces body-own androgen production and AR antagonists inhibit androgen-mediated activation of AR. Here, molecular mechanisms are described that review knowledge about tumor cells escape therapy by developing bypass mechanisms of AR-signaling. This includes genomic and non-genomic signaling. Deciphering the involved molecules that mediate castration and drug resistance will provide the basis of potential novel drug targets that may be used in addition to AR inhibition as combinatory treatment.

**Abstract:**

Androgen receptor (AR) is a main driver of prostate cancer (PCa) growth and progression as well as the key drug target. Appropriate PCa treatments differ depending on the stage of cancer at diagnosis. Although androgen deprivation therapy (ADT) of PCa is initially effective, eventually tumors develop resistance to the drug within 2–3 years of treatment onset leading to castration resistant PCa (CRPC). Castration resistance is usually mediated by reactivation of AR signaling. Eventually, PCa develops additional resistance towards treatment with AR antagonists that occur regularly, also mostly due to bypass mechanisms that activate AR signaling. This tumor evolution with selection upon therapy is presumably based on a high degree of tumor heterogenicity and plasticity that allows PCa cells to proliferate and develop adaptive signaling to the treatment and evolve pathways in therapy resistance, including resistance to chemotherapy. The therapy-resistant PCa phenotype is associated with more aggressiveness and increased metastatic ability. By far, drug resistance remains a major cause of PCa treatment failure and lethality. In this review, various acquired and intrinsic mechanisms that are AR‑dependent and contribute to PCa drug resistance will be discussed.

## 1. Introduction

### 1.1. Role of AR in PCa and its Inhibition of AR in PCa Therapy

Prostate cancer (PCa) is the most commonly diagnosed non-cutaneous cancer and the second leading cause of cancer deaths in males in Western countries [1]. PCa growth is regulated by the androgen receptor (AR), activated upon androgen binding [2]. AR is not only essential for the development of the normal prostate gland but also promotes the progression of PCa. Since most PCa at early diagnosis are androgen-dependent (also termed castration-sensitive PCa (CSPC)) [3], inhibition of AR signaling by ADT is the first-line treatment. Although the original form of ADT is the surgical castration, currently the chemical castration with ADT drugs, such as luteinizing hormone-releasing hormone (LHRH) agonist or antagonists that reduce serum testosterone to castration levels, is used [4]. However, unfortunately, within 30 months of persistent ADT, all PCa patients eventually develop resistance to castration therapy [5]. This stage is referred to as castration-resistant PCa (CRPC), where patients display increased serum PSA levels in serum despite ADT, suggesting a dysregulated activation of AR signaling [6].

**Figure 1:**
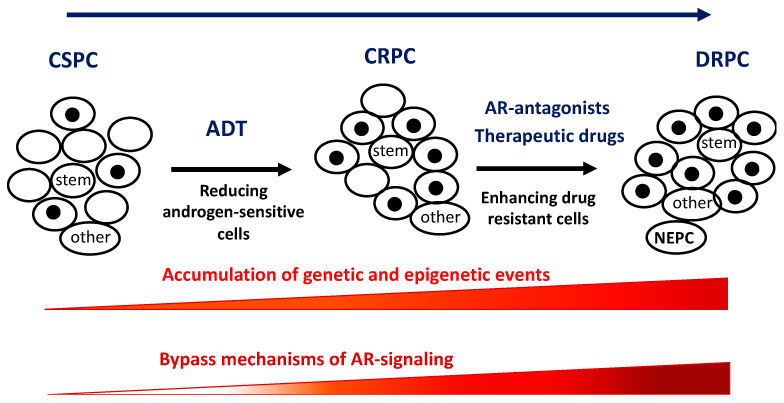
Schematic view of prostate cancer tumor evolution upon therapy. In general, due to accumulation of mutations, a tumor is composed of many cancer cell types leading to tumor cell heterogeneity. Cancer consists also of cancer stem cells (stem) and other non-cancerous cells (other, including cancer -associated fibroblasts and immune cells). Androgen-deprivation therapy (ADT) is mostly successful inhibiting the growth of androgen-sensitive PCa cells. However, castration-resistant cells may be selected by the treatment and accumulate. Treatment with AR antagonists and other therapeutic drugs, including chemotherapy and radiation, might select for drug resistant PCa cells leading to a more aggressive tumor (such as NEPC). Associated with the tumor evolution, during tumorigenesis PCa develops a variety of androgen bypass signaling.

Currently, CRPC patients are additionally treated with AR-antagonists such as enzalutamide (Enz) and darolutamide (ODM-201) to fully block the AR-axis. The use of these AR-antagonists shows long terms benefits, including significantly lowering serum PSA level, decreases the risk of metastasis and prolonged overall survival in CRPC patients [7,8]. However, resistance occurs eventually also against AR antagonist treatment resulting in drug resistance PCa (DRPC), mostly associated with activation of the AR-axis. The reactivation of AR signaling after long-term inhibition of AR-axis is partly because PCa cells slowly develop multiple adaptive mechanisms of resistance in response to chronic exposure to low testosterone and AR antagonist environment. However, AR remains an essential driver in this progression [6,9,10]. Thus, tumor evolution seems to be associated with an adaptive response of AR signaling bypassing ADT and antagonist activity and perhaps selecting for a more aggressive drug resistant CRPC (Figure 1). Until recently, metastatic CRPC (mCRPC) lacked effective treatment options. In this review, the molecular mechanisms leading to therapy resistance against ADT and antagonists and AR bypass mechanisms as well as adaptive signaling of PCa will be discussed.

Mechanistically, AR antagonists can induce cellular senescence in PCa cells in vitro as well as ex vivo in patients PCa samples. This indicates that AR antagonists do not completely inhibit the AR rather promote the induction of an AR-dependent cellular senescence pathway [11,12,13]. Although cellular senescence induces an irreversible cell cycle arrest being in general advantageous for PCa therapy, senescent cells show a senescence associated secretory phenotype that might induce cell proliferation within the tumor microenvironment including in neighboring non-senescent PCa cells. Inducing cell proliferation of non-senescent PCa through senescent cells might be one mechanism of tumor evolution towards antagonist resistance. Therefore, senolytic compounds will be beneficial as co-treatment [13].

### 1.2. Genomic Action of AR

Classically, androgens, preferably dihydrotestosterone (DHT), bind to the AR, induce a conformational change that leads to dissociation from heat shock factors. This allows dimerization and nuclear translocation of AR to regulate the target genes. The activated AR acts as a transcription factor and suppresses or activates directly androgen-responsive target genes such as prostate-specific antigen (PSA) and many other genes involved in the differentiation and proliferation of prostatic cells [14]. AR antagonists, also known as anti-androgens, antagonize the action of androgen through different mechanisms. They may compete for the ligand binding by blocking the ligand-binding domain (LBD) of the AR. AR antagonists prevent to fully activate the AR since the binding of AR antagonists induces a different conformation of the receptor compared to agonists. Some AR antagonists reduce the translocation of the ligand-bound-AR complex into the nucleus and/or inhibit the recruitment of transcriptional co-activators resulting in inhibition of the AR-mediated gene transactivation [15] and finally, leading to repression of PCa growth [11,16,17]. Compared to the previous generation of AR antagonists, the development of the next generation of AR antagonists has significantly improved the life span and overall survival of PCa patients [18].

### 1.3. AR Gene Amplification, Mutations and AR Splice Variants

The most common documented genomic aberration in CRPC, with around 60% of tumors, is AR gene amplification or mutation, leading to increased AR activity [18]. AR overexpression results from different mechanisms such as gene body amplification increased histone acetylation/ phosphorylation at AR enhancers, overexpression of AR co-regulators, or enhanced protein stability of AR [19,20]. Several studies have shown that gene amplification leads to DRPC and in addition to ADT failure with increased risk of PCa metastasis [2]. Notably, the isolated circulating tumor cells (CTC) from mCRPC patients with abiraterone- or Enz-resistance showed gene amplification of AR (AR gain) in 50% of patients [21].

It is widely accepted that mutations of AR in PCa under selection by anti-androgen treatment are one of the main reasons for DRPC [22,23]. Most acquired point mutations map to the LBD of AR, altering the conformation of the ligand-binding site so that some AR antagonists can act as potent AR agonists and lead to drug resistance [22,24,25]. It is nearly 30 years that the AR T877A point mutation (previously known as 868 Thr to Ala mutation) in the LBD is known for resistance to hydroxyflutamide treatment, a first-generation anti-androgen [25,26]. This mutation renders hydroxyflutamide from a potent antagonist into a potent agonist. Similarly, the point mutations AR F876L and F876L/T877A switch Enz from AR antagonist to an AR agonist [23]. It has also been reported that the AR point mutations W741C, T877A, W741L, W741C/T877A, F876L, F876L/T877A, and L701H can switch Bicalutamide (Bic), a first generation anti-androgen, from an AR antagonist to a potent AR agonist leading to reactivation of AR signaling in PCa under therapy. However, the underlying molecular mechanism of the switch is poorly understood [23]. 

Another adaptive mechanism that bypasses ADT and leads to therapy resistance is the expression of AR splice variants. Approximately one-third of Enz or abiraterone treated patients express truncated receptors arising from AR splice variants (AR-Vs) [27]. Enz treatment leads to enhanced AR-V7 expression that eventually makes PCa cells resistant to Enz. Knockdown of AR-V7 restores the sensitivity of CRPC cells to Enz, pinpointing the role of AR-V7 in CRPC drug resistance [28]. AR-Vs with a deleted LBD lack a binding site for first- and second-generation AR antagonists in order to mediate antagonism. Deletion of the AR LBD leads to a constitutively active AR. It was shown that part of AR signaling in these PCa cells is changed, indicating a ligand-independent AR activation. Of note, AR‑V7 binds together with the full-length AR and represses a higher number of genes that it activates [29]. Thus, AR-V7 action is still under investigation but represents one mechanism to bypass antagonist treatment and is a trigger for an androgen-independent pathway for activation of AR signaling. 

CRPC patients who express AR-V7 have both a shorter progression-free survival and overall survival. These patients fail mostly to respond to Enz or abiraterone treatment, and thereby the suggested treatment is those alternative drugs that target the N-terminal domain (NTD) [27], such as the EPI compounds [30]. EPI compounds are AR‑antagonists that irreversibly bind to the AR NTD at the transcription activation unit 5 and inhibit AR-mediated transactivation. These compounds inhibit both ligand-activated AR and ligand‑independent activation of AR, including the AR splice variants [31,32]. Therefore, EPI compounds act independently of the AR-LBD deletions or mutations and may be used for the treatment of CRPC dependent on AR-Vs. 

So far, several EPI-derived compounds have been designed, analyzed on the molecular level and included in clinical trials. A new derivative of EPI is EPI-506 or ralaniten acetate that is used in a clinical trial for mCRPC patients as an oral soft gel capsule (NCT02606123). In this study, different concentrations (80–1800 mg) of EPI-506 were used, while only high doses (>1280 mg) showed a meaningful decrease in PSA level for a short period [33,34]. This clinical trial was stopped due to poor pharmacokinetics properties besides upregulation of UGT2B enzymes (UDP-glucuronosyl-transferase), which are involved in glucuronidation. A new development to identify AR antagonists that target AR NTD is another analog of ralaniten, EPI-045, being however resistant to glucuronidation, which might optimize the clinical response to AR-NTD inhibitors [35]. This compound has not yet been used in clinical trial. Another analog of ralaniten, EPI-7170, was used as a monotherapy and in combination with Enz for PCa cells that express AR-V7 and are resistant to Enz. This compound inhibits transcription of both full-length AR and AR-V7 target genes. The combinatory treatment resulted in stronger inhibition of proliferation rather than monotherapy [28]. Notably, a phase I trial of mCRPC patients has been started with a new EPI called EPI-7386 (NCT04421222) since the beginning of 2020. 

In CRPC a specific AR enhancer located 650 kb centromeric to the AR gene body was identified to be activated, led to gain of function probably due to the selective pressure exerted by anti-androgen treatment. This AR enhancer gene is either co-amplified with the AR gene in CRPC or even amplified at a higher rate. Further, it was detected that AR enhancer is enriched with H3K27 acetylation [36]. Another group detected the same enhancer to be duplicated in mCRPC and post-progression biopsy samples [37]. Taken together, the AR enhancer amplification can contribute to the desensitization of PCa cells to hormone therapy.

### 1.4. Co-Regulators of AR 

The AR transcriptional activity is regulated by a series of co-regulator complexes such as chromatin remodelers, co-activators and co-repressors that are co-recruited by the AR to chromatin by binding directly or in a protein complex to AR to regulate gene activity. A battery of different co-activators and co-repressor are known that interact with the AR [38]. In general, it is suggested that the balance of co-activator and co-repressors is required for fine tuning AR-mediated transcription. A misbalance of the co-regulators can lead to higher AR activity in PCa and less active antagonism by anti-androgens [38,39,40,41].

The binding of AR to androgen response elements (ARE) itself can be regulated by diverse factors. The prostate-specific tumor suppressor gene *CHD1* encodes an ATP-dependent chromatin remodeler. CHD1 regulates AR occupancy at a subset of AR binding sites of lineage-specific enhancers. *CHD1* loss alters AR binding at lineage-specific enhancers and modulates distinct transcriptional programs to drive prostate tumorigenesis [42]. It has recently been shown that the loss of *CHD1* in vivo is the basis of a heterogeneous drug resistance mechanisms to Enz [43].

Another AR co-regulator and chromatin pioneering factor is homeobox B13, HOXB13, which interacts with AR to bind to AR target loci. HOXB13 is a homeodomain transcription factor that is involved in the development of normal prostate, as well as the formation and maintenance of prostate cancer [44]. Interestingly, in different stages of PCa progression, HOXB13 seems to change its expression pattern. Analysis of PCa TCGA datasets revealed that HOXB13 mRNA level in PCa is higher than in normal prostate tissues [45]. Moreover, analysis of transcriptome expression from several databases revealed that the expression of HOXB13 is elevated during the progression of primary PCa to CRPC [46]. This is supported by another study showing that the induced expression of HOXB13 promotes androgen-independent growth of androgen-sensitive LNCaP cells by activating E2F signaling [47]. Mechanistically, HOXB13 mediates also the oncogenic function of AR-V7, which is strongly associated with a CRPC phenotype [48]. Notably, HOXB13 interacts with AR-V7 to a much higher degree than with the full-length AR across the CRPC genome [49]. These findings could explain its role in DRPC resistance towards anti-androgen treatment. Interestingly however, in the transformation from CPRC to the more aggressive neuroendocrine PCa (NEPC), the expression of HOXB13 is lowered or lost [45,46], indicating a central role for HOXB13 in tumorigenic evolution.

Alongside with HOXB13 other pioneering factors such as FOXA1 and GATA2 act as chromatin factors at enhancer sites to regulate on one hand the expression of AR and on the other hand the activity of AR and AR-V7 at chromatin in mCRPC. FOXA1 induces open chromatin conformation to allow the binding of other transcription factors [50]. This pioneering transcription factor enhances AR transactivation and CRPC progression [21]. Of note, FOXA1 locus is one of the most frequent mutated sites in both primary PCa and metastatic PCa. Mutations in both cis-regulatory elements and coding sequence cause serious functional alterations [50]. Truncated FOXA1 protein shows higher DNA binding affinity than the wild type FOXA1, leading to activation of WNT signaling and progression of cancer [51]. In line with this, isolated circulating tumor cells (CTC) from patients with abiraterone- or Enz-resistant metastasis showed gene amplification of FOXA1 in more than 30% of CRPC patients [21], pinpointing the critical role of FOXA1 in emerging DRPC.

Also, GATA2 promotes AR activity and enhances progression of androgen dependent PCa to CRPC status. High GATA2 expression correlated with more aggressiveness of PCa [52]. Higher expression of GATA2 in the CRPC cell line ARCaPM leads to increased expression of insulin-like growth factor (IGF2) and thereby chemotherapy resistance [53]. ACK1 is an oncogenic tyrosine kinase that interacts directly with AR and enhances AR DNA binding and thereby AR transcriptional activity through phosphorylation of AR at Tyr267 and Tyr363 sites, which promotes androgen independent growth of PCa in the LAPC-4 cell line [54,55]. Recently it has been revealed that ACK1 has in addition, epigenetic regulatory function [19]. The ACK1-AR complex is recruited to AR enhancer and epigenetically enhances AR expression by phosphorylating histone H4 at Tyr88, leading to upregulation of AR expression. This drives the androgen-independent program in CRPC. ACK1-mediated phosphorylation of histone H4 seems to be a key mechanism in the emergence of DRPC in response to AR-antagonists. This epigenetic mark has emerged as an important mechanism for CRPC drug resistance [19].

Another epigenetics regulator of AR is enhancer of zeste homolog 2 (EZH2), which is a member of the polycomb group protein. [56]. Of note, it has been observed that injecting astemizole in CRPC mouse xenografts significantly represses interaction of EZH2 with AR and inhibits tumor growth [57]. LSD1 is another epigenetic regulator of AR that facilitates AR-dependent transcriptional activity [58]. Luciferase reporter assay have shown that chemical inhibition of LSD1 reduces activation of both wild type AR and AR-V7 in CRPC 22RV1cells [59].

Another AR-coactivator is CREB5, being amplified and overexpressed in CRPC, and associates with resistant PCa. In a recent clinical study, studying more than 1000 PCa patients under Enz treatment has shown that the expression of the oncoprotein CREB5 was abnormally high in ADT resistant tumors, which is due to an increased copy number of CREB5. High expression of CREB5 enhances the proliferation of PCa cells under ADT condition and mediates also resistance to Enz treatment. Therefore, drugs targeting CREB5 might be useful in DRPC therapy [60]. 

Also, AR co-repressors may mediate castration resistance. The AR co repressor LCoR is recruited to AR in the presence of androgen. This recruitment of LCoR to androgen-bound AR inhibits AR action associated with inhibition of tumor progression in xenograft tumors of CRPC cells [61]. The overexpression or activation of Src protein kinase has been shown in metastatic PCa and CRPC [62]. Interestingly, the activated Src inhibits the gene-silencing function of LCoR in CRPC cells [61,]. Thus, it suggests that the activation of the membrane-associated Src non receptor tyrosine kinase negatively regulates LCoR co-repressor activity and thereby promotes tumor development.

### 1.5. Non-Genomic Action of AR

Besides the genomic action of AR to regulate gene expression by recruitment to chromatin, the non-genomic action of AR includes the regulation of several signaling pathways in the cytoplasm, including membrane-bound protein receptors [63].

It is known that not all androgen-activated AR translocates into the nucleus. Androgen-activated AR interacts in the cytosol with other factors at non-genomic level. This interaction leads on one hand to modulation of signal transduction pathways and on the other hand, to phosphorylation of AR. Factors that interact with AR include the non-receptor tyrosine kinase Src, AKT and PKA. In addition, AR interacts with PTEN-PI3K, and the mTOR-autophagy pathways. These pathways cross-regulate each other. They may therefore contribute to drug resistance and promotion of PCa progression [63,64]. Various pre-clinical studies showed as an example that the activation of PI3K is associated with the development of CRPC [65,66]. Thus, in a clinical view, understanding the non-genomic AR signaling pathways is essential since they may be responsible for the potential resistance to current anti-androgen therapy [63,64].

Preclinical studies showed that nerve growth factor (NGF) signaling enhances proliferation and metastasis in PCa. NGF binds to tropomyosin receptor kinase A (TrkA), which in turn activates the Ras/MAPK and AKT pathways [67]. In LNCaP cells, the AR associates with TrkA by treatment with NGF or androgen, although androgen exhibits a stronger effect. This suggests a reciprocal induction of a crosstalk between AR and TrkA to promote PCa proliferation and migration. A TrkA inhibitor represses the mitogenic effect of androgen. Notably, the AR antagonist bicalutamide inhibits NGF signaling. These findings postulate a possible advantage of combination treatment to inhibit both AR and TrkA [68]. Notably, the same group showed that in C4-2B, a CRPC and more aggressive cell line compared to LNCaP, the NGF induced proliferation of PCa cells seems not dependent on AR [67]. Thus, the NGF/TrkA interaction with AR might be specific for less advanced and less aggressive types of PCa.

A further important aspect which promotes PCa progression and contributes to drug resistance is the PCa microenvironment. The tumor microenvironment consists of stromal and inflammatory cells in addition to cancer-associated fibroblasts (CAFs). CAFs lead to the reorganization of structure and composition of the connective tissue, as well as enhanced tumor growth, invasiveness, and drug resistance of PCa. Hence, CAFs promote PCa progression. In the presence of androgen, the AR co-localizes with the scaffold protein filamin A (FilA) in fibroblasts. The AR/FilA complex results in CAFs moving towards PCa cells in 2D and 3D culture by increasing migration and invasiveness. An AR-derived staple peptide, Rh-2025u, was developed to disrupt the AR/FilA complex. The disruption inhibited migration and invasion. This peptide also impaired the AR induced metalloprotease cascade and thereby inhibiting remodeling of extracellular matrix, implying to be an interesting new therapeutic drug useful in combination with AR inhibition [69].

### 1.6. Bypass-Mechanisms of Aberrant Androgen Action to Activate AR Signaling Activation

In addition to the expression of AR splice variants that lack the AR LBD, other mechanisms of alternative activation of AR signaling have been described. In line with the interference of PI3K/AKT signaling with AR, PTEN gene mutations are common in PCa cancerogenesis, with loss of PTEN being identified in many metastatic PCa. PTEN is a tumor suppressor that inhibits the PI3K/AKT pathway. This augments the upregulation of the PI3K-AKT pathway in PCa and especially in mCRPC. First studies focused on blocking PI3K/AKT pathways as a possible therapeutic approach to treat PCa, since PI3K, through basal activation of AKT, has been strongly related to PCa growth and progression. Pharmacological inhibition of PI3K increased AR protein levels and thereby activating HER3-mediated AR-related gene expression [70]. This result provides a possible explanation for transient or even failing treatments targeting PI3K/AKT pathway in PCa. Also, inhibition of AKT lead to similar results by reducing FOXO inhibition, leading to FOXO mediated AR expression [70,71]. Furthermore, inhibition of AR by the antagonist Enz resulted in downregulation of FKBP5, which is a chaperone for AKT phosphatase PHLPP, and thereby increasing phosphorylation of AKT [72]. This suggests that AR inhibition by antagonists may result in the activation of AKT signaling. Another study also suggests that there is an interaction of AR signaling with PI3K-AKT pathway to promoting progression of PCa to CRPC [73]. This mechanism might be a possible explanation for how AR antagonism through activation of AKT signaling promotes DRPC.

Multiple studies, therefore, investigated the possible benefits of combined treatment compared to single-agent therapy. Treating xenograft mouse models of androgen insensitive LNCaP with AZD5363, an AKT inhibitor leads to transient inhibition of tumor growth and PSA stabilization but followed eventually by resistance to AZD5363. However, combined treatment of a CRPC mouse model with the AKT inhibitor, AZD5363, and the AR antagonist Bic prolonged disease stabilization without signs of drug resistance [74]. Another study showed that treatment of both CRPC (C4-2) or LNCaP cells with AZD5363 combined with Enz resulted in decreased cell growth via induction of cell cycle arrest and apoptosis. Moreover, the combinatory treatment of LNCaP xenograft model of CRPC significantly reduced tumor size without relapse compared to monotherapy with either Enz or AZD5363 [66], indicating that a combinatory treatment by inhibition of both the AKT and AR pathways could be beneficial.

Src kinase also emerges as a major player in the resistance of PCa. Src belongs to the family of non-tyrosine kinase family (SFK). Src expression is often correlated with malignant tumors and has been targeted in many therapies in PCa [75]. On one hand, Src activates AKT and on the other hand Src interacts with AR leading to its phosphorylation. Thereby, Src kinase induces phosphorylation of AR directly and indirectly [76]. Further, Src kinase can induce the expression of genes involved in proliferation and survival in PCa cells in an androgen-independent manner, as well as certain Src specific genes that correlate with early PCa metastasis onset and poor survival [77]. Additionally, it has been reported that Src kinase activity, which is mediated by non-genomic AR action, is associated with PCa cell invasion [78]. Thus, further supporting the hypothesis of a Src-AR complex that activates AR and Src-dependent activation of PCa growth-promoting genes.

Mechanistically, it is suggested that the Src kinase-mediated phosphorylation of AR at Tyr267 in the NTD, harboring a potent AR transactivation domain, as well as Tyr551 and Tyr552 in the hinge region, harboring the AR NLS, promotes both the nuclear translocation and transactivation by AR. Inhibition of Src kinase with PP2 was able to reduce AR translocation [79]. Pre-clinical in vivo mouse studies revealed that Src activation inhibits AR interaction with co repressors like LCoR and promotes the binding of AR to its target genes [61,80]. However, clinical trials using dasatinib, a tyrosine kinase inhibitor, in combination with abiraterone for treatment of mCRPC did not significantly prolong progression-free survival. Therefore, the study was terminated early, although the study cohort was not complete [81]. Interestingly, Src can form a ternary complex with AR and the estrogen receptor and thereby, activate Erk-2 resulting in increased proliferation of PCa cells [82,83]. Also, this ternary complex regulates EGF-induced EGFR leading to enhanced DNA synthesis and mitogenesis. Toremifene, a selective estrogen receptor modulator showed beneficial effects in PCa treatment [82,83].

Noteworthy, an interesting link between Src and mutation in the tumor suppressor genes BRCA1 and BRCA2 was shown. It was reported that in mCRPC with loss of BRCA1/2 heterozygosity Src signaling is enriched, therefore being more sensitive to Src inhibitors relative to mCRPC with wildtype BRCA2. Furthermore, Src knockdown in BRCA-null PCa cells enhanced PARPi sensitivity and indicated that Src activation might be a mechanism for PARPi resistance. This is supported by the findings using a combined treatment of a Src inhibitor with a PARPi inhibitor revealing a synergistic effect to inhibit proliferation of BRAC2-null mCRPC. Thus, inhibition of both Src and PARP is a promising new approach to treating mCRPC [84].

### 1.7. Intratumoral and Alternative Androgen Biosynthesis

ADT and AR-antagonist treatment may also fail due to intratumoral androgen production and therefore can lead to castration resistance. It was shown that intratumoral testosterone and DHT levels in mCRPC patients were significantly higher than in the control group [85]. Drug resistance can result from adaptive intratumoral androgen biosynthesis, which can be caused by gene alterations. For example, the 367T mutation in the enzyme 3βHSD1 augments the enzymatic activity resulting in increased DHT synthesis in CRPC [86]. Also, it has been reported that copy number and expression of HSD17B3 is increased in mCRPC [87]. Also, an increased expression of the steroid-5α-reductase isoenzyme-1 (SRD5A1) associates with emergence of CRPC. The enhanced expression of SADAR1 increases production of DHT from testosterone [88]. Notably, inhibiting the other key enzyme CYP17A1 by the small molecule VT-464 inhibits androgen biosynthesis and CRPC tumor growth [89]. Another enzyme implicated in testosterone synthesis is HSD17B6, which was also shown as a bypass mechanism to be upregulated under ADT and thereby can cause resistance to ADT [90]. An increase in intratumoral androgen biosynthesis may also lead to reduced efficacy of AR antagonists and thus lead to DRPC. To inhibit the intratumoral androgen biosynthesis, the CRPC patients are treated with abiraterone [91]. The use of abiraterone in chemotherapy-naïve CRPC patients meaningfully improved the progression-free survival [92]. However, the use of this inhibitor in CRPC patients also faced failure. 

### 1.8. Role of Autophagy in AR-Dependent Drug Resistance

The AKT-mTOR pathway regulates autophagy, which is suggested to be also a crucial pro-survival mechanism for CRPC [93,94]. Under metabolic stress, such as androgen deprivation, autophagy can be an adaptive response to sustain cell survival and induce DRPC [95].

It was shown that CRPC cells can survive PI3K inhibition. The suggested underlying mechanism is that AR increases the expression of Integrin α6β1 and indirectly Bnip3. Active AR-Integrin signaling suppresses apoptosis and induces Bnip3-mediated autophagy and mitophagy. Notably, inhibition of integrin α6β1 re-sensitized PTEN-negative CRPC to PI3K inhibitors. Hence, this might explain the lack of successful clinical trials of PI3K inhibitors for AR-dependent CRPC and unfold on the other hand a new approach to therapy [96].

A mechanism of therapy resistance by AR antagonists may be explained by the findings that Enz and Bic induce autophagy leading to a pro-survival response in PCa cells [93,97]. Apaluatmide, another AR antagonist used in the treatment of CRPC, also increases levels of pro-survival autophagy in LNCaP. Interestingly, the combination of AR inhibition by apalutamide and an autophagy inhibitor enhances apoptotic cell death in PCa cells compared to the single treatments [98]. Thus, enhanced autophagy may be induced by AR antagonists as adaptive signaling to overcome growth inhibition and to promote resistance to therapy.

### 1.9. AR-Bypass Mechanisms to Mediate Castration Resistance

Therapy resistance of PCa is not only caused by AR-dependent mechanisms but also by AR-independent mechanisms, still activating a part of AR signaling. A short overview of possible pathways, which results in primary and acquired resistance, is described briefly in the following. The glucocorticoid receptor (GR, NR3C1) is also a nuclear steroid receptor and closely related to the AR. GR becomes increasingly expressed in primary PCa tissue after long-term treatment with Enz [99]. Thereby confirming another study, which showed the overexpression of GR could result in clinical resistance to Enz [100]. However, it has been shown that inhibition of BET impairs GR signaling and resensitizes drug-resistant tumors to Enz, highlighting the role of epigenetic modulator in PCa therapy to overcome Enz resistance [101].

Mechanistically, GR may bind an overlapping set of AR target genes [102,103,104] and may thereby bypass AR blockage but allows the activation of a part of the AR transcriptome landscape. Dexamethasone, a GR agonist, has been shown to decrease the GR protein level in AR-null PCa cells (DU145 and HH870), which resulted in inhibited growth. Surprisingly, the observed inhibition of growth was enhanced with Dexamethasone combined with Enzalutamide. The response of these AR-null PCa cells to Enz is unclear but might suggest off-target binding of Enz in high concentration (25µM) to GR, since decreased growth was also shown with Enz alone. In the same study mifepristone, a GR and progesterone receptor antagonist also inhibited the growth of AR null PCa cells (DU145 and PC3 cell line). Interestingly, all these cell lines have a moderate to high expression of GR in common. Taken together, GR can activate a part of AR-signaling. Inhibition of GR might be an interesting target in treating PCa and especially enhancing the efficacy of mifepristone alone or in combination with an AR antagonist could be of interest [105].

GR expression and activity is also associated PCa cells with resistance to radiation. After radiotherapy, resistant cells have constitutive activated GR and decreased expression of AR. In radioresistant PCa, results suggest that radiation-induced GR through increased cAMP levels. Survival is promoted by GR in androgen-dependent PCa, but not in CRPC [106]. Instead, GR enhances the metastatic potential of CRPC. This offers a new and interesting target for treating radioresistant PCa [106]. Therefore, highly specific GR and progesterone receptor agonists or antagonists are required to overcome AR independent therapy resistance.

Combined ADT with AR-antagonists may also cause resistance and lead to the development of neuroendocrine PCa (NEPC), presumably due to selective pressure from ADT and AR antagonist treatment [107]. NEPC is a very aggressive form of PCa and has a higher ability to metastasize [10,108]. One key driver of NEPC is N-Myc, encoded by *MYCN*, which is required for PCa tumor maintenance [109]. The conversion of CRPC to NEPC associates with *MYCN* amplification or N-Myc overexpression [58]. It has been shown that overexpression of N-Myc and AKT1 in mice drives NEPC progression from human prostate epithelium [109]. RNA-seq data and immunohistochemical staining of the NEPC and CRPC patient’s tumors show that overexpression of N-Myc, presumably due to *MYCN* amplification, is associated with shorter survival [110].

N-Myc overexpression in C4-2 cell line confers resistance to Enz by increasing ATM kinase activity, which promotes migration and invasion of PCa cells [111]. Mechanistically, N-Myc is phosphorylated by Akt in the cytoplasm, which then translocates into the nucleus [110,112]. This is further supported by another study in that overexpression of N-Myc in LNCaP and C4-2 decreases expression of AR and its target gene KLK3/PSA [111]. However, the preliminary studies of NEPC patients show attenuated AR signaling in tissue samples [113], some recent clinical data show evidence of the persistent AR expression in a subset of NEPC patients [114,115]. This suggests a possible alternative role of AR in those NEPC cells, which is still unclear.

ONECUT2 is a transcription factor identified as a master regulator of AR networks in mCRPC. ONECUT2 suppresses the AR transcriptional program by direct regulation of AR target genes and the AR licensing factor FOXA [116]. ONECUT2 activates genes associated with neural differentiation and progression to lethal disease. ONECUT2 regulates hypoxia through activating SMAD3 in NEPC [117]. The median time from the first diagnosis to the progression of NEPC phenotype is around 20 months [118]. NEPC tumorigenesis is still poorly understood hence complicating possible treatment courses. Since the transformation of PCa to NEPC is thought to develop a resistant mechanism to AR‑directed therapies, N-Myc and ONECUT2 are suggested as a promising target in CRPC therapy.

p53 interferes with AR signaling through binding to the AR promoter and suppressing AR expression. Enhanced p53 protein level negatively regulates the AR mRNA and protein levels in LNCaP cells. Knock down of p53 enhances AR protein expression in LNCaP cells [119]. Mutations in p53 occur in most PCa tumors and mostly cause changes in p53 protein conformation, leading to reduced or loss of p53 function. Overexpression of p53 mutants leads to chemotherapy resistance and drug resistance (including resistance to cisplatin, temozolomide, doxorubicin, gemcitabine, tamoxifen and cetuximab) in primary PCa and metastatic PCa [120]. Since p53 enhances PCa cells apoptosis, stabilizing p53 protein and thereby enhancing p53-mediated apoptosis in PCa cells promotes the effectiveness of chemotherapy. However, p53 protein level can be reduced by inhibitors such as MDM2. MDM2 reduces p53 protein level through ubiquitination and subsequent proteasomal degradation is MDM2 (murine double minute 2) [120,121]. Interestingly, MDM2 gene amplification has been found in PCa. Therefore, stabilization of the MDM2-p53 complex is an important therapeutic target for stabilizing p53 in cancers. In line with this, inhibition MDM2 stabilizes p53 and thereby sensitizes PCa cells to ADT and radiation therapy or combination therapy [122].

Another AR-bypass pathway is the activation of the AKT-mTOR pathway by p66Shc, which is an oxidase previously shown to promote androgen-independent cell growth through generation of reactive oxygen species (ROS) and being elevated in PCa tumor samples. P66Shc is also known as a sensor for oxidative stress-induced apoptosis and as a longevity protein in mammals. Interestingly, in cancer p66Shc seems to activate AKT, mTOR, ERK, and Rac1 [123]. Since p66Shc regulates PCa cell migration through ROS- mediated activation of migration-associated proteins, p66Shc might be involved in the development and progression of androgen-sensitive PCa to CRPC by enhancing the production of oxidant species as well as enhancing metastatic potential [124].

### 1.10. Non‑Coding RNAs and Drug Resistance in PCa

MicroRNAs (miRNAs) are endogenous small non-coding RNAs that act as a tumor suppressor or oncogene post-transcriptionally. Emerging evidence has discovered the importance of aberrant expression of miRNAs that are involved in the development of CRPC. Deregulated miRNA mediate drug resistance through different mechanisms such as reducing apoptosis by downregulating E2F1, enhancing CSC properties to facilitate tumorigenicity and chemoresistance, promoting the EMT process to increase PCa aggressiveness. For example, upregulation of miR 221/-222 via targeting p27/Kip1, HECTD2, and RAB1A are responsible for 90% of CRPC [125].

Cisplatin is commonly used as a chemotherapy agent for the treatment of cancer. Chemotherapy of patients with cisplatin is initially effective, but patients eventually develop resistance [126]. The lncRNA PCa-associated transcript 1 (PCAT-1) that mediates cisplatin resistance is suggested as a marker for prostate cancer [127]. PCAT-1 is an oncogenic lncRNA that positively regulates c-Myc [128] and negatively regulates BRCA2 (tumor suppressor) [127]. miR-205 binds to 3′UTR of AR and inhibits AR at both transcription and translation levels in both LNCaP and 22RV1 cells [129]. miR-31 directly targets AR and upregulation of miR-31 inhibits the expression of AR at protein and RNA levels and suppresses PCa growth in vivo [130]. The gene expression of both miR-205 and miR-31 is down regulated (suppressed) in advanced PCa compared to normal PCa cells. The low expression of these two miR-RNAs stabilizes AR protein and makes PCa cells resistant to apoptosis and might be the reason for PCa resistance to cisplatin and docetaxel. Therefore, a low level of miR-31 and miR-205 correlates to cisplatin and docetaxel resistance [131]. Also, a link between non-coding RNAs and the AKT-mTOR in PCa pathway has been described. SNHG1 is up-regulated in PCa tissue and binds to EZH2, also mostly upregulated in PCa. Knock-down experiments suggest that the proliferation, migration and invasion of LNCaP and PC3 cells were significantly reduced. On a molecular level, SNHG1 upregulates both Wnt/β-catenin and the PI3K-AKT-mTOR pathway in cell lines and in mouse xenografts [132]. Thus, non-coding RNAs seem also to interfere with AR signaling to mediate PCa tumorigenesis or therapy resistance.

### 1.11. Role of Cytokines and Growth Factors in AR Signaling

In CRPC, the overexpression of cytokines such as IL-6 is known to promote tumor progression and drug resistance [133,134,135]. Through the autocrine signaling of IL-6, PCa cells activate constitutively STAT3. Subsequently, STAT3 activity increases AR recruitment to AR target genes such as to the PSA promoter. It is suggested that STAT3 pathway is essential to drive PCa progression to mCRPC by in part by reactivating the AR pathway [136,137]. In addition, STAT3 activation may promote Enz resistance of CRPC. Because of that, inhibition of STAT3 has been an interesting treatment for potential therapy [137,138,139]. It was reported that inhibition of STAT3 results in decreased growth of Enz-resistant cells. In line with this, niclosamide has been reported to block STAT3 signaling by inhibiting its phosphorylation. Furthermore, Liu et al. showed that niclosamide resensitized Enz resistant cells are supporting a role for IL-6-STAT3 pathway in Enz-resistance [140]. 

The IL-6 signaling pathway is seemingly important in the regulation of growth and drug resistance of PCa cells and it helps cancer cell survival. A phase I study with patients scheduled for radical prostatectomy received either siltuximab or no drug. Tumor samples of siltuximab treated patients showed a decrease in [141] phosphorylated STAT3 and p44/p42 MAPK, which leads to a downregulation of target genes of IL-6 signaling and key enzymes of androgen signaling [142,143]. A phase II study by SWOG conducted with Docetaxel- pretreated patients with CRPC resulting in a stable disease by RECIST criteria (Response Evaluation Criteria in Solid Tumors) in 23% of patients (7 out of 31 patients). After the treatment, an increase of IL-6 was observed, probably due to the accumulation of stable IL-6/anti-IL-6 monoclonal antibody complexes. This might be a possible explanation for the lack of clinical benefit of targeting IL-6 [144,145]. Also, in a randomized phase II study, a combination of mitoxantrone/prednisone and siltuxumab was not associated with a clinical improvement as compared with chemotherapy alone [141]. 

Another pathway that involves STAT3 is an aberrant TGF-β pathway that plays a major function in the occurrence of DRPC and induction of epithelial-mesenchymal transition (EMT) because members of TGF-β family induce the expression of Snail [146]. Snail is a transcription factor one key regulator of EMT that represses the expression of E cadherin, an important event in EMT [147], a biological process of the morphological transition of epithelial cells becoming mesenchymal cells. This process includes enhanced migratory capacity, elevated resistance to apoptosis, and increased production of ECM components [148]. Since ADT can induce EMT, the metastatic potential of PCa will eventually be enhanced. It is assumed that the molecular basis is an aberrant TGF-β pathway [146]. An ongoing Phase II trial (NCT02452008) is analyzing the efficacy of galunisertib, a TGF-β inhibitor, in combination with Enz. Interestingly, EMT can be inhibited by metformin treatment and restore sensitivity to Enz-resistant mice xenografts. Metformin seems to block EMT by targeting the TGF-β/STAT3 axis [149]. This might be a promising new approach to target and prevent EMT and associated metastasis in PCa.

Moreover, reduced or loss of AR expression results in activation of IL-6 signaling, which activates STAT3. Activation of STAT3 is associated with enhanced Sox2, CD44, Nanog, Musashi-1, and integrin α2β1 that are stemness regulators. Thus, this may lead to a cancer stem cell (CSC) phenotype. Mounting the CSC repertoire results then in an unwanted sustained malignancy of human PCa tumors and is suggested to be responsible for the emergence of DRPC and tumor relapse [150]. 

Epidermal growth factor receptor (EGFR) is involved in regulating cellular growth and function. The EGF ligand causes activation of EGFR by autophosphorylation, which leads in multiple downstream signaling [151]. The mRNA and protein level of EGFR in PCa is higher compared to the normal prostate. In PCa, the activated AR upregulates EGFR gene expression. In turn, activation of EGFR by its ligand, EGF, signals downstream to multiple pathways such as JAK/STAT, PI3K/AKT and MAPK pathways leading to an increasing AR activity. This ultimately enhances androgen-independent tumor growth by altering proliferation, migration and survival [151]. EGFR is known to be involved in PCa progression [152]. One mechanism of docetaxel resistance in CRPC is through EGFR signaling. Upon docetaxel treatment, PCa cells increase EGFR expression and activation. EGFR induces AKT-dependent ABCB1 expression. Geftinib, an EGFR inhibitor, and knockdown of EGFR could resensitize docetaxel resistant cell to docetaxel [153]. Inhibition of EGFR signaling with Spautin-1 proved to potently decrease the growth of PCa xenografts by inducing apoptosis in PCa cells. Combined treatment of PCa xenografts with Spautin-1 and ENZ had a synergistic inhibitory effect on tumor growth [154]. This finding provides an interesting therapeutic approach by targeting AR and EGFR signaling in stratified PCa with high active EGFR.

## 2. Conclusions

**Figure 2:**
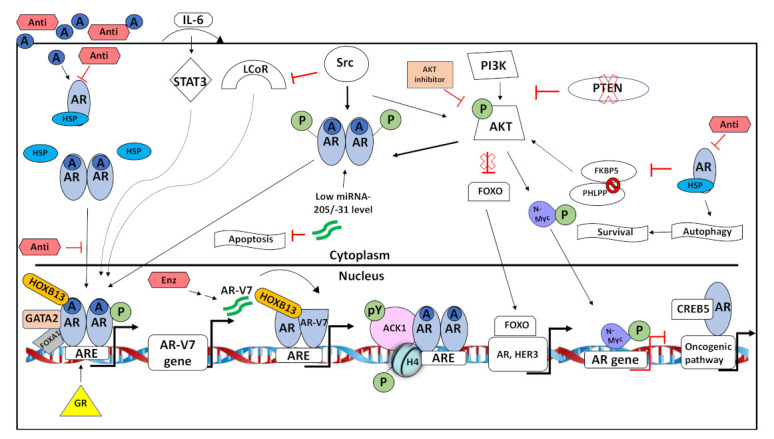
Summary of some adaptive AR signaling pathways active in DRPC. Androgens and AR-antagonists (Anti) diffuse into PCa cells. Androgens bind and activate the androgen receptor (AR) in the cytoplasm. The AR dissociates from HSP, forms a dimer and translocates into the nucleus. Antagonists inhibit androgen-activated AR by various biochemical mechanisms and block activation of AR signaling. At chromatin, co-regulators such as pioneering factors, co-activators and co-repressors modulate transcriptional activity of AR at androgen response elements (ARE). Adaptive signaling that lead to CRPC includes the upregulation of glucocorticoid receptor (GR) that can bind to AREs and can activate AR signaling despite AR inhibition. Through autocrine signaling IL-6 activates STAT3 that in turn phosphorylates AR and thereby enhancing AR-mediated transactivation. The second-generation AR antagonist Enzalutamide (Enz) increases the expression of AR-V7 variant associated with aggressive PCa and dimerizes with AR to change AR transcriptome landscape. Activation of Src kinase phosphorylates AR and AKT. Both Src and AKT interact with AR and phosphorylate AR to enhance its nuclear translocation. Additionally, Src kinase inhibits LCoR and thereby inactivating its function as co-repressor of AR. Low expression of miRNA-205/-31 stabilizes AR protein and blocks apoptosis. Phosphorylated ACK1/AR complex phosphorylates chromatin and increases accessibility to target genes. PTEN loss results in active PI3K/AKT signaling. Active AKT phosphorylates AR and N-myc. Phosphorylated AR translocates to the nucleus and induces expression of ARE. N-Myc represses AR gene expression. AKT inhibition blocks repression of FOXO by AKT. Hence, FOXO is active and induces expression of genes such as AR and HER3. Inhibition of AR by anti-androgens downregulates FKBP5 expression. Therefore, FKBP5 cannot act as a chaperone of PHLPP leading to the hyperphosphorylation of AKT. AR inhibition by AR further induces pro-survival autophagy. CREB5 forms a complex with AR, resulting into expression of genes involved in oncogenic pathways.

The AR is a critical genomic and non-genomic factor in PCa pathogenesis. Adaptive AR signaling and occurrence of therapy resistant signaling are selected through therapy leading to CRPC and DRPC with unwanted further PCa progression. It emerges that many different adaptive and bypass pathways can lead to DRPC, which emphasizes the need for personalized medicine (Figure 2). AR-dependent mechanisms mediating drug resistance in prostate cancer can be at the level of resistance towards ADT, subsequently also resistance to AR antagonist treatment, towards inhibitors of androgen biosynthesis, or other pathways. It emerges that many of the resistance pathways overcome the AR as a drug target either by overactivation of AR or by overactivation of AR signaling, such as the PI3K-Src-AKT-mTOR-autophagy, STAT3, or GR pathway. Defining these pathways in drug resistance shall lead to combinatory treatment options by targeting the AR together with drugs that block specific bypass signaling pathways.

## Abbreviations

AR	androgen receptor
AR-V7	an AR splice variant
ADT	androgen deprivation therapy
ARE	androgen response element
Bic	bicalutamide
CAF	cancer-associated fibroblasts
CRPC	castration-resistant prostate cancer
CTC	circulating tumor cells
CSC	cancer stem cell
DRPC	drug resistance Pca
DHT	dihydrotestosterone
EGFR	epidermal growth factor receptor
Enz	enzalutamide
EMT	epithelial-mesenchymal transition
GR	glucocorticoid receptor
LCoR	ligand-dependent co-repressor
mCRPC	metastatic CRPC
NLS	nuclear localization signal
NEPC	neuroendocrine prostate cancer
LBD	ligand-binding domain
NTD	amino-terminal domain
ODM	darolutamide
Pca	prostate cancer
PSA	prostate specific antigen
SFK	Src family of kinases
